# Liquid biopsy approaches and immunotherapy in colorectal cancer for precision medicine: Are we there yet?

**DOI:** 10.3389/fonc.2022.1023565

**Published:** 2023-01-06

**Authors:** Sheefa Mirza, Kinjal Bhadresha, Muhammed Jameel Mughal, Michelle McCabe, Reza Shahbazi, Paul Ruff, Clement Penny

**Affiliations:** ^1^ Department of Internal Medicine, Faculty of Health Sciences, University of the Witwatersrand, Johannesburg, South Africa; ^2^ Department of Internal Medicine, Common Epithelial Cancer Research Centre, Faculty of Health Sciences, University of the Witwatersrand, Johannesburg, South Africa; ^3^ Hematology/Oncology Division, School of Medicine, Indiana University, Indianapolis, IN, United States; ^4^ Department of Biochemistry and Molecular Medicine, School of Medicine and Health Science, The George Washington University, Washington, DC, United States; ^5^ Department of Anatomical Pathology, School of Pathology, Faculty of Health Sciences, University of the Witwatersrand, Parktown, Johannesburg, South Africa

**Keywords:** liquid biopsy, neoantigen, immune cells, exosomes, colorectal cancer, mitochondrial DNA, circulating cancer associated fibroblasts (cCAFs)

## Abstract

Colorectal cancer (CRC) is the second leading cause of cancer-related deaths globally, with nearly half of patients detected in the advanced stages. This is due to the fact that symptoms associated with CRC often do not appear until the cancer has reached an advanced stage. This suggests that CRC is a cancer with a slow progression, making it curable and preventive if detected in its early stage. Therefore, there is an urgent clinical need to improve CRC early detection and personalize therapy for patients with this cancer. Recently, liquid biopsy as a non-invasive or nominally invasive approach has attracted considerable interest for its real-time disease monitoring capability through repeated sample analysis. Several studies in CRC have revealed the potential for liquid biopsy application in a real clinical setting using circulating RNA/miRNA, circulating tumor cells (CTCs), exosomes, etc. However, Liquid biopsy still remains a challenge since there are currently no promising results with high specificity and specificity that might be employed as optimal circulatory biomarkers. Therefore, in this review, we conferred the plausible role of less explored liquid biopsy components like mitochondrial DNA (mtDNA), organoid model of CTCs, and circulating cancer-associated fibroblasts (cCAFs); which may allow researchers to develop improved strategies to unravel unfulfilled clinical requirements in CRC patients. Moreover, we have also discussed immunotherapy approaches to improve the prognosis of MSI (Microsatellite Instability) CRC patients using neoantigens and immune cells in the tumor microenvironment (TME) as a liquid biopsy approach in detail.

## Introduction

Colorectal cancer (CRC) is third in terms of the most common (6.1%) and second in terms of deadly (9.2%) disease worldwide. It is estimated that by the year 2035, the total number of deaths from rectal and colon cancer will increase by 60% and 71.5%, respectively. Overall survival (OS) 5 years after primary diagnosis in stage I–II is 87-90%, decreasing to 68–72% in stage III, and futher lowering to 11–14% in stage IV metastatic CRC (mCRC) (1–5). Today therapeutic algorithms for CRC contain endoscopic and surgical resection, systemic adjuvant chemotherapy, radiation therapy, targeted therapy, and immunotherapy (6, 7). Due to the poor response of numerous colorectal patients to existing therapeutic approaches and since CRC survival is highly dependent on primary diagnosis and early treatment, a known significant biomarker that can predict the beneficial response as early as possible is immediately required. To date, tissue biopsy is one of the best standard options for tumor identification. Though, the main drawback is that it is problematic to screen disease development over frequent biopsies due to recurrent injury and poor patient compliance. Tissue removal also carries hazards, and it is unapproachable for some cancer cases (8, 9). Also, biopsy has some significant boundaries: it is invasive, expensive, painful has technical boundaries related to tumor location, and is not effective in pointing to tumor cells subpopulations (10–12). Indeed, there is a critical need to find a minimally invasive or non-invasive method to screen the high-risk population and detect CRC presence in asymptomatic patients at an earlier and curable stage.

The awareness of liquid biopsy is that of identifying circulating biomarkers to distinguish cancer cells released from the primary tumor and/or metastasis sites (13–15). The meaning of ‘liquid biopsy’ describes the importance of identifying cancer-derived biomarkers in blood or other body fluids, such as stool, saliva, cerebrospinal fluid or urine (16–22). The very noteworthy targeted constituent studied in liquid biopsy is circulating tumor DNA (ctDNA), circulating tumor cells (CTC), circulating tumor RNAs, and exosomes (23–27). Although they are the most studied component for liquid biopsy, CTCs alone cannot be considered as a clinical diagnostic tool due to the debate over their clinical utility (28). However, it has been reported that tumor cells communicate not only with additional malignant cells, but also with the constituent of the tumor microenvironment (TME), suggesting their stability in circulation is highly reliable on TME (29, 30). So, here we hypothesized that CTC research should be commenced concurrently with other TME components, such as, cancer associated fibroblasts (CAFs), various immune cells, extra-cellular vesicles (EVs) etc. Furthermore, another noninvasive approach being studied is the use of ctDNA, exosomal miRNAs, and proteomics; which though in primary stages, needs to be elucidated in-depth. Additionally, we have highlighted the benefits of immunotherapy treatment for MSI-high (MSI-H) CRC patients and use of neoantigens and immune cells as a liquid biopsy approach for better prognosis.

**Figure 1:**
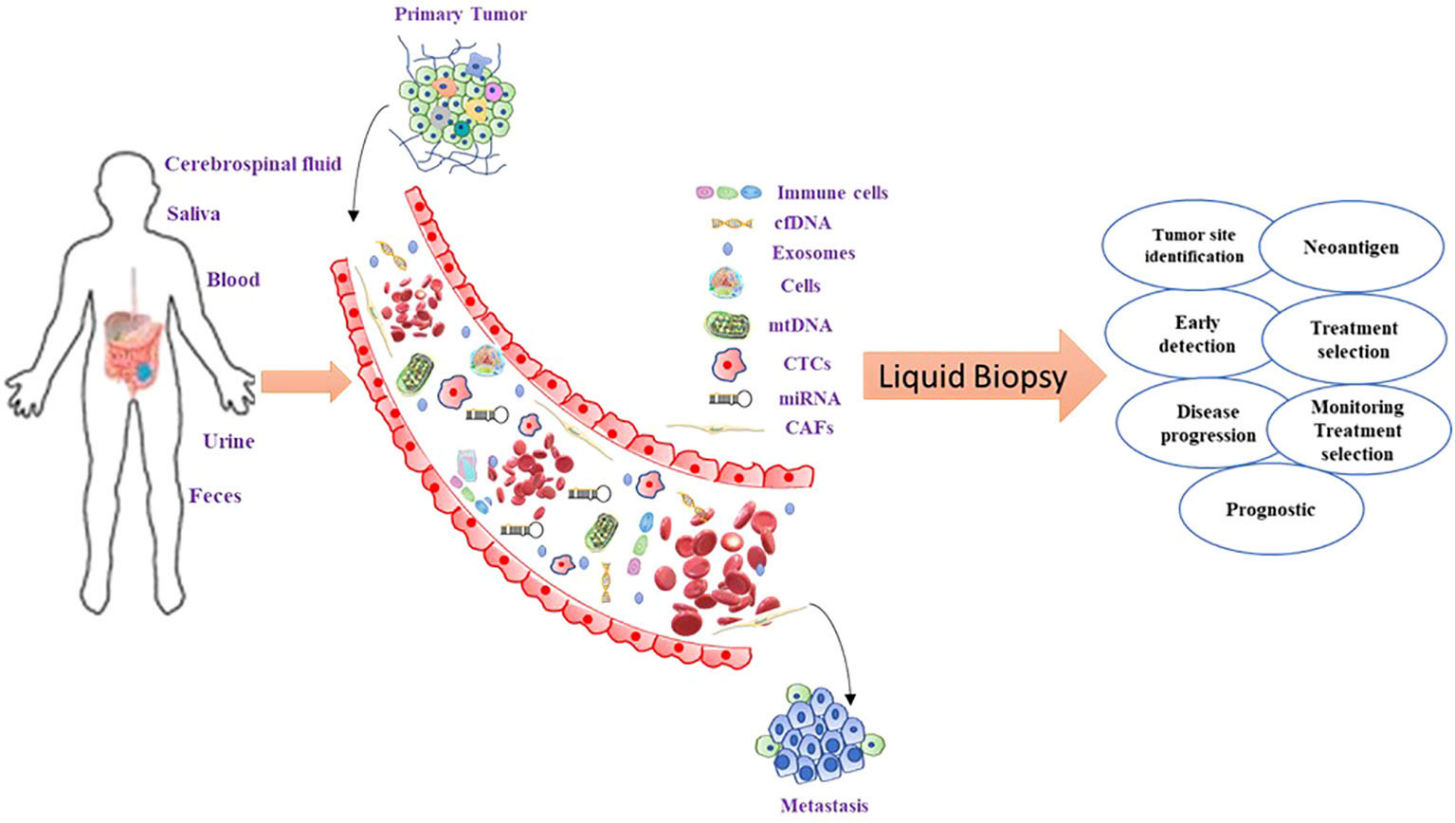
Liquid biopsy components in CRC and their clinical utility. CTCs are shedded from the tumor into the blood vessels where they can release their components: nucleic acids and exosomes with tumor-specific cargo material. For the analysis of these molecules, blood can be taken out, and plasma or serum further processed for the extraction of the desired constituents. From the blood circulation, these molecules can be filtered into saliva and urine which can also be collected and further analyzed. Each of these constituents delivers one or more levels of tumor information. The quantity of the concentration of single proteins or panels including numerous tumor proteins is the present gold standard in cancer management.

Overall in this review, we have described the concept of liquid biopsy and its applications in the management of CRC patients (**Figure 1**). Furthermore, we have highlighted the role of less explored components, such as organoid models of CTC, immune cells in TME, mitochondrial DNA (mtDNA), and neoantigens in the liquid biopsy approach. These approaches could be used noninvasively to gain knowledge about molecular characterization and the mechanism of disease progression in CRC.

## Tumor-microenvironment components

The awareness of the tumor microenvironment (TME) has been proposed more than one hundred years ago. In the meantime, cancer research has discovered many several noteworthy roles of TME components not only in cancer metastasis, but also in cancer metabolism and development (31–33). TME consists of a web of cancer cells, stromal cells, immune cells, CAFs, exosomes, and extracellular matrix. In this composition, immune cells and stromal cells are the two major non-tumor cell types in addition to tumor cells (34). Interestingly, TME Web found it possible to achieve immune organization of cancers concerning prognosis, chemotherapy, and prediction of immunotherapy response (35–37). In the current scenario, several studies on cancer have shown that TME meaningfully affects cancer cell proliferation and development and recommends potential worth in the diagnosis and prediction of cancer prognosis (38–40). In addition, it has been suggested that TME is highly affected by the development of CRC (41–43). Furthermore, the TME components play significant roles in defining CRC with poor prediction and immune escape (44, 45). Together, the significant function of TME in the development and metastasis of CRCs and the investigation of the essential molecular mechanism that makes the interaction between the transformation of TME and the progression of CRCs have fascinated important considerations over the past era. But until now, a comprehensive understanding of the TME in CRC development and metastasis has yet to be discovered.

### Circulating tumor cells and circulating cancer-associated fibroblasts: Symbiotic siblings and potential drug targets

**Figure 2:**
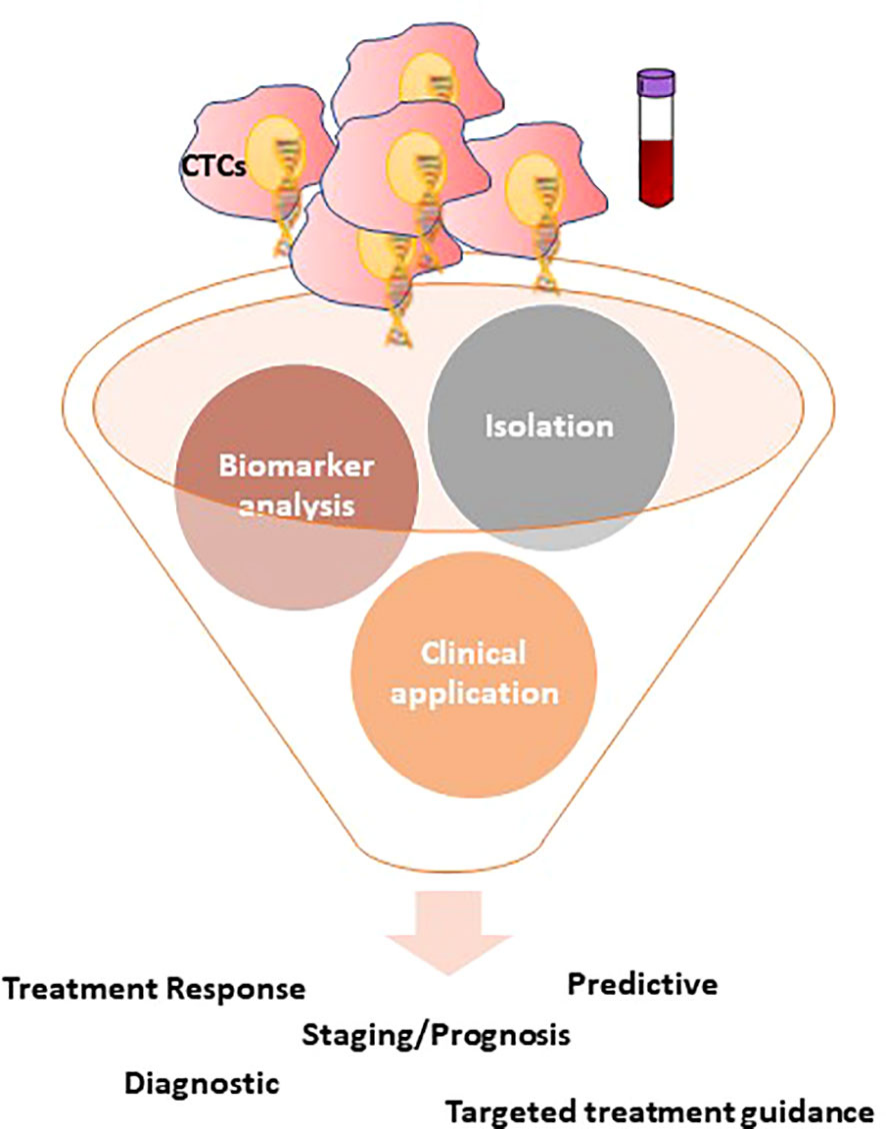
Overview of the CTCs detection technologies and the potential clinical applications of CTCs in CRC. CTCs isolation can usually be divided into two groups: physical isolation designed to exploit the differing physical belongings of blood components, such as size, deformity, and charge; and biological isolation, often utilizing antibody-based capture methods to enrich CTCs or deplete various blood cells. Following isolation, CTCs are open to a variety of downstream applications, focusing primarily on one of three categories: enumeration, characterization, and expansion.

CTCs are the representative of the cancer cells detached from the primary tumor which enter into the circulatory system (blood, lymphatic system) to cause metastasis (46, 47). Undoubtedly, CTCs have been used as a dynamic component of liquid biopsies to investigate the presence of residual cancer cells, track treatment response, and prediction of disease recurrence, which is suggestive of the fact that CTCs could play a critical role in the early diagnosis and prognosis of various cancers, including the development of personalized therapeutic options (48–53). Compared to other cancer biomarkers, CTCs are cancer cells that could carry biological and molecular evidence of cancer cells that supports single-cell analysis and directly provide information about ongoing alterations in cancer cells at all different stages of disease progression (54–56). Based on the evidence, CTCs have a favorable role in early prediction, therapeutic observation, and disease progression and would be a significant drug target for various cancers (57–60). The existence of clusters of CTCs has been reported during the last decade, and several groups have described the clinical relevance of CTC clusters. Although the prognostic value of CTC has been well validated, limitations are preventing the use of CTC enumeration in routine clinical practice concerning the use of CTCs either as a clinical marker for early cancer detection, or as a surrogate endpoint in interventional studies (61). These limitations include uncertainty about the specificity of CTC detection assays and justifiable concerns that CTC detection alone may be misleading or inadequate, especially when applied in the early detection of metastases. Additional biomarker assays can enhance the specificity and broaden the application of “liquid biopsies” in early cancer detection, monitoring disease progression, and determining response to therapy (**Figure 2**). To relate to a single cancer cell, CTC clusters are comparatively low and rare in circulation, but reveal noteworthy, better resistance to apoptosis and additional metastatic potential (62–64). Likewise, research on clusters of CTCs in the peripheral blood of patients with CRC has revealed that the clusters of CTCs are not malignant, but relatively tumor-derived endothelial cells connected to the vascular features; particularly, the separation and counting of these clusters of CTCs can distinguish between healthy individuals and patients with early-stage CRC with a high degree of precision (IIa) (48, 65).

**Figure 3:**
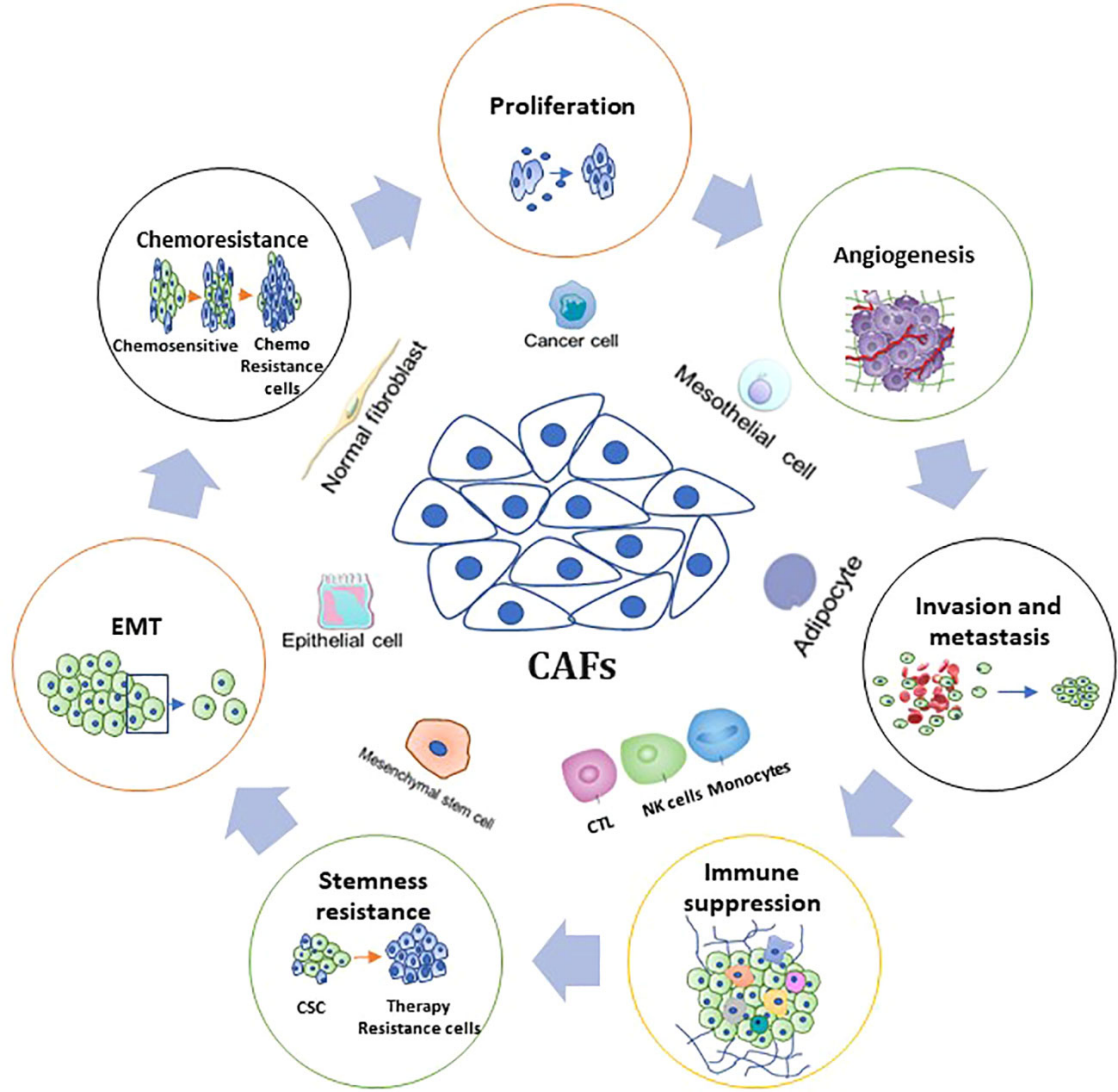
Fundamental Functions and associated mechanisms of CAFs in CRC hallmarks. The figure shows the role of CAFs in CRC biology, including tumorigenesis, proliferation, angiogenesis, invasion and metastasis, stemness, therapy resistance, and tumor immunity.

Because CTCs can be detected in the peripheral blood of cancer patients, it follows that a “liquid biopsy” to detect tumor components in blood will not only contain tumor cells but will also contain other cellular components of TME. Cancer-associated fibroblasts (CAFs) – responsible for cancer cell proliferation, migration, invasion, drug resistance, and other important biological processes through secretion of cytokines, chemokines, and growth factors - are a heterogeneous population and an essential component of cells in TME (**Figure 3**) (66). Various studies have revealed their inevitable role in the regulation of almost all hallmarks of cancer, resulting in tumor progression and metastasis (67–69). According to Dr. Paget’s seed and soil theory, the seed has been repeatedly studied as cancer stem cells (CSCs), resident? cancer cells, and more recently as CTCs; whereas soil is represented as the TME (70). It is very well known that in the CTC population, most CTCs die at an early stage when they enter the circulation due to the collective effects of environmental and mechanical factors, for example, oxidative and sheer stress and immune response (48, 71). Consequently, only a few drug-resistant cells can escape and spread by undergoing a series of modifications to survive the changing environment. By looking at this theory, it is proposed that caves form clusters with CTCs to provide a suitable TME to CTCs and/or CTSCs (circulating tumor stem cells) for their persistence during metastasis in the circulation.

**Figure 4:**
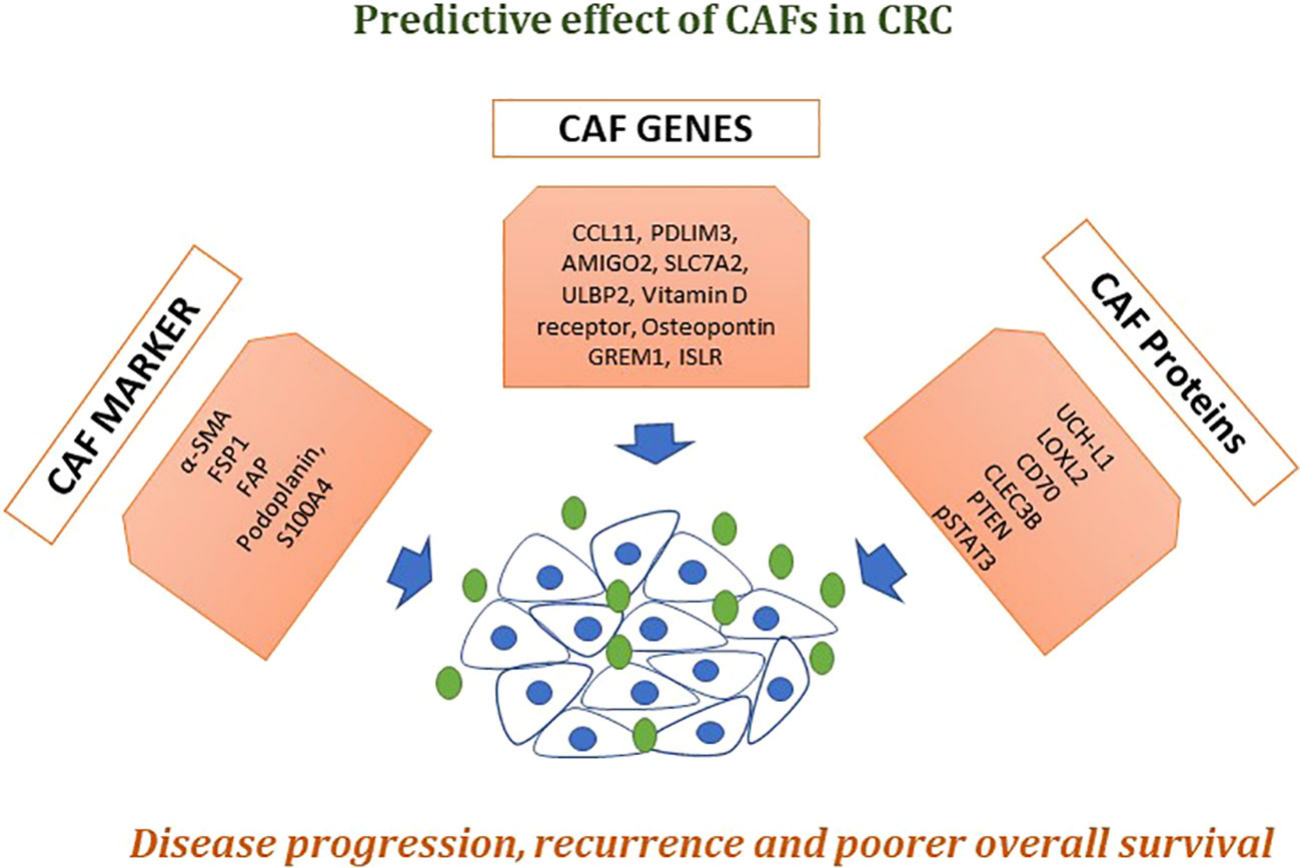
Effects of CAFs in CRC. A numeral of biomarkers that are extremely expressed in CAFs, like α-SMA, fibroblast activation protein alpha (FAP), fibroblast-specific protein 1 (FSP-1), platelet-derived growth factor receptor-α (PDGFRα) and PDGFRβ have now been commonly used to classify or isolate CAFs from the pool of fibroblasts present in the whole body. Described genes and proteins showed poorer disease progression, recurrence-free survival, and overall survival. Taken together, these markers could be used as liquid biopsy approach for early detection and treatment prognosis in CRC patients.

In CRC, various studies have reported clinical applications of CTC for early diagnosis, prognosis, and treatment monitoring using different techniques (48). In addition to this, recent studies revealed the importance of the CTC cell line in classifying cancer-associated proteins (neoantigens) and pathways connected to cancer cell stemness and metastasis, as well as in assessing anticancer drug sensitivity (72–76). Agarwal et al. identified clusters of cCAFs/CTCs and discovered that the cumulative number of these clusters is associated with cancer growth and metastasis (77). Although these studies have shown the presence of CAFs outside of the primary tumor site or metastatic lesions, there has been little direct evidence showing the presence of CAFs in the circulation of cancer patients in a clinical setting. In addition to this, several biomarkers, genes, and proteins have been extremely highly expressed in CAFs and also have poorer disease progression and overall survival in CRC (**Figure 4**). To date, the importance and use of CTCs in clinical setting for CRC is increasingly being established (**Table 1**) (26, 78–88), but the low population and vast heterogeneity of CTCs in addition to the progress of diagnosis and analysis approaches have few common approvals to use CTCs as a new biomarker. Thus, impeding cCAF/CTC complex formation or dismantling them, as well as clusters with other types of cells, may open new frontiers for controlling cancer or preventing metastasis.

**Table 1:** List of CTCs biomarkers and its clinical use in CRC.

Biomarkers	Methods	Clinical use
EpCAM	CellSearchTM, CanPatrolTM	Predictive and prognostic
CEA	RT-PCR	Prognostic
CK19	RT-PCR, CK19-Epispot	Prognostic
CD133	Drug sensitivity analysis of CTC lines	Prognostic
CKs	RT-PCR	Prognostic
VIM	CanPatrolTM	Prognostic
TWIST1	CanPatrolTM	Prognostic
CD26	Drug sensitivity analysis of CTC lines	Prognostic
CD44v6	Drug sensitivity analysis of CTC lines	Prognostic
KRAS	Label-free Vortex technology	Prognostic
BRIEF	Label-free Vortex technology	Prognostic
PI3KCA	Label-free Vortex technology	Prognostic
AKT2	CanPatrolTM	Prognostic
SNAI1	CanPatrolTM	Prognostic

### Exosomal miRNAs/ctDNA/cfDNA

A stimulating realm of tumor research has advanced over the past decade by concentrating on extracellular vesicles (EVs), known as exosomes, to answer pivotal challenges around therapeutics, diagnosis, and prevention. Exosomes are known as vesicles formed *via* the endocytic pathway and ranging in size from 30-140nm in diameter. As a new significant focus on the enigma of cancer, exosomes signify a noteworthy characteristic of biological signaling between cells and are also used as novel biomarker identification strategies (89–91). In addition, numerous studies have discovered that body fluids harbor abundant quantities of EVs, the constituent’s quantity of which varies based on the physiological or pathological state of an individual (92, 93). These diverse populations of extracellular? vehicles transfer detailed cargo such as miRNA, proteins, and lipids from one cell to another to stimulate a specific response. Exosomes can be found in all body fluids and can be detected in liquid biopsies (94). This section focuses primarily on exosomes containing miRNAs, proteins, and mRNA that appear to be consistently altered in patients with CRC. To date, there are only a minority of publications aimed at understanding exosomes in relation to CRC. EVs released from CRC cells can reveal vital evidence about significant molecules and signaling pathways involved in the growth and development of CRC (95, 96). Thus, the existence of tumor-derived EVs in circulating body fluids makes them prospective innovative biomarkers for early prognosis, diagnosis, and prediction of CRC cancer.

**Figure 5:**
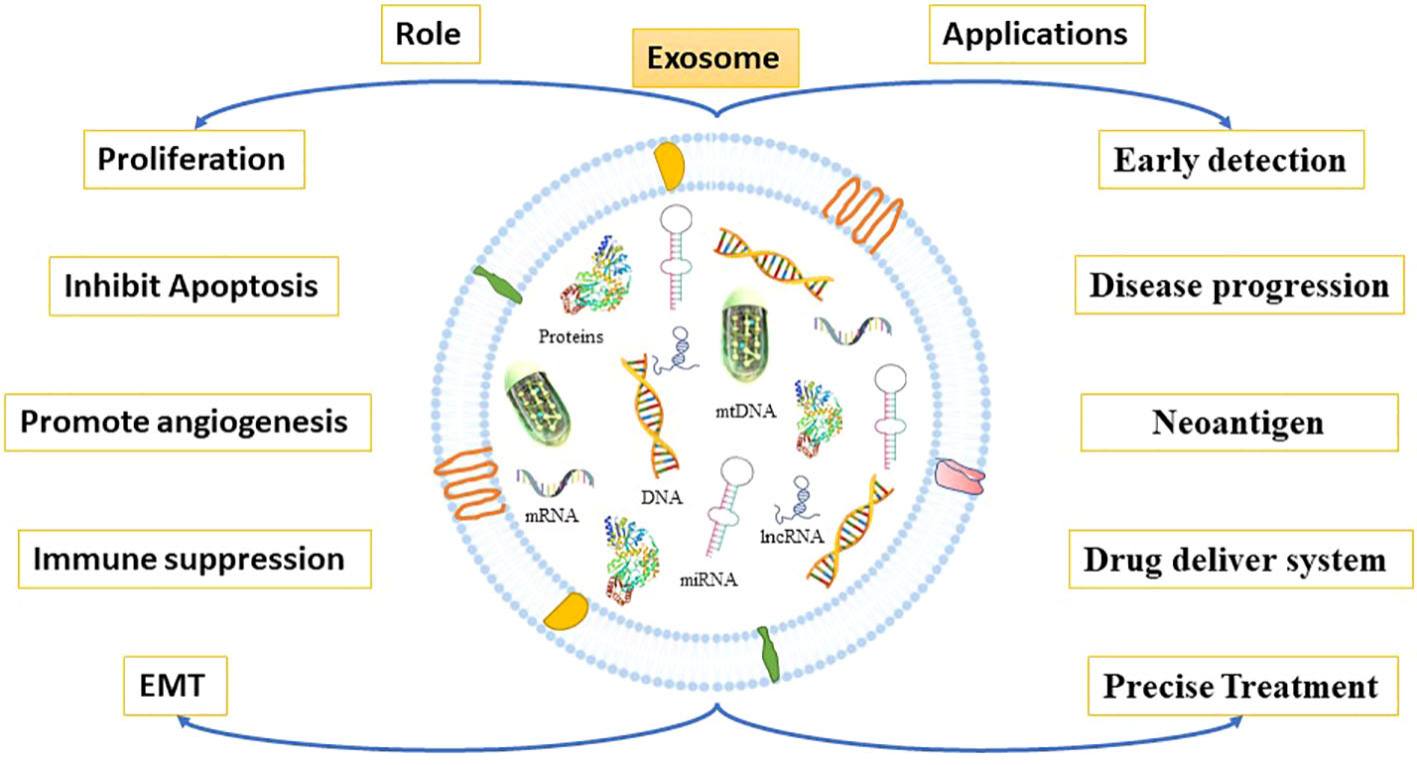
Roles and application of exosomes. Tumor-derived exosomes promote cancer growth and metastasis. Through multiple mechanisms, they participate in cancer growth and metastasis by reshaping TME resulting into EMT, cell proliferation, apoptosis inhibition, immunosuppression, and angiogenesis. Exosomes derived from cancer cells are enriched with proteins, mRNA, miRNA, lncRNA, DNA etc. that are more abundant in cancer cells than in normal cells. Thus, exosomes may be used as biomarkers for cancer diagnosis, prediction, and treatment.

Exosomes have a prominent role in cell proliferation, metastasis, and epithelial-to-mesenchymal transition (EMT), as well as by supporting the angiogenic switch and the remodeling of the extracellular matrix (ECM) in CRC (97, 98) (**Figure 5**). In recent research, it was observed that CRC cells released more exosomes in hypoxic conditions (99, 100). Besides, these exosomes encourage cell proliferation *via* shortening the mitosis period and triggering STAT3 signaling in CRC (95, 101). Furthermore, Mulvey et al. demonstrated that co-culture of the CRC HCT116 cell line exosomes with normal colon cells can increase its clonogenicity (102). Numerous cellular components in exosomes have been reported that could contribute to CRC metastasis through various molecular mechanisms. A recent research report suggested that glycoprotein A repetition-dominant (GARP) knockdown Mesenchymal stroma/stem-like cells prevent the cell proliferation and invasion of mouse colorectal cancer cells through exosomes (103).

Exosomal miRNAs have been significantly concerned in several exosome-mediated biological functions in cell-cell communication in numerous cancers including CRC (104, 105). MiR-21-5p and miR-155-5p have been revealed to be highly expressed in macrophage-derived M2 exosomes, which facilitated the migration and invasion of CRC (106, 107). In addition to this, it also observed that exosomes from bone marrow-derived mesenchymal stem cells (BMSCs) can inspire stem cell-like features of colorectal cancer through miR-142-3p (108). In addition, CAFs, TAM, and MSC exosome proteins are also significant mediators of cancer and TME regulation. Gang, N, and his team used proteomic analysis of CAFs and serum-derived exosomes that have recognized QSOX1 as a biomarker for the early prediction and detection of CRC non-invasively (109). Current research also described novel types of RNAs, such as Piwi-interacting RNA (piRNA) and tRNA-derived small RNA (tsRNA), along with miRNA, lncRNA, and cicrRNA (110, 111). There has only been limited research into the current existence and role of these types of non-coding RNAs in CRC exosomes. Thus, even though the therapeutic approach of exosomes has revealed countless application scenarios in colorectal cancers, many problems remain before we can routinely use exosomes in the clinical treatment of CRC.

In most solid tumors, CAFs are the significant cellular components of the TME (112). CAF-derived exosomes could stimulate neoplastic angiogenesis and cancer cell growth in CRC. Furthermore, these can also activate cancer cell dedifferentiation through the Wnt signaling pathway, therefore increasing the chemical resistance of CRC (95, 113). Compared to RNA and protein, there is little research on exosomal DNA. In previous research, it was observed that the gDNA from exosomes is widely used in liquid biopsy, and it has a great impact on tumor immunity and metabolism (114–118). The KRAS and BRAF mutation was identified in serum exosomes of patients with CRC with greater sensitivity and specificity (119, 120). Furthermore, it was also revealed that exosome gDNA plays a significant role in immunity in CRC patients (117). Current research studies have revealed that the number of exosomes in the body fluid of CRC patients is markedly higher compared to healthy controls (98). Hence, the studies on CRC exosomes must be encouraged due to this increased presence of CRC exosomes that can likely be used during cancer treatment.

Another promising biomarker that has established noteworthy consideration in the current era is circulating tumor DNA (ctDNA). ctDNA comprises DNA fragments that are released by fragmenting tumor cells into the blood circulation and in principle should have genetic and epigenetic alterations identical to the cancer cells they initiated from (121, 122). Numerous types of DNA modifications have been noticed with adaptable frequency in the ctDNA of patients with CRC. The revealing of mutant DNA in plasma or serum from a CRC patient has been associated with diagnosis, prognosis, and therapeutic response in numerous reports (123). Furthermore, in CRC patients *KRAS* mutations in ctDNA have been identified in different stages, with the highest level found in the more advanced stage (124–126). Furthermore, recent studies found that ctDNA was detected postoperatively in approximately 5% to 30% of patients with stage II to III colon cancer and has established a strong prognostic capacity in numerous observational studies (127). Since the last decade, in CRC, the introduction of next-generation sequencing (NGS) technology has led to the discovery of ctDNA in plasma, which is an encouraging practice (128, 128). Some research reports revealed that ctDNA methylation has a notable sensitivity compared to traditional serum tumor markers in patients with initial-stage CRC and is a significant biomarker for the diagnosis of CRC (129–131). Currently, personalized immunotherapy based on neoantigens requires tissue samples to obtain accurate evidence of somatic genomic modifications in individual cancer patients. Although it is from time to time problematic to obtain many tumor tissues; consequently, the development of ctDNA analysis could be significant in the enlargement of neoantigen-based treatment, even though it is still puzzling. Together, current potential clinical trials with ctDNA focus on the diagnosis, surveillance, and prognosis of CRC. With the rapid progress of research technology, liquid biopsies will play a crucial role in the diagnosis and treatment of CRC. In **Tables 2**, **3** (132–179), we have listed circulating miRNAs, lncRNAs, circ-RNAs and proteins as diagnostic biomarkers in CRC patients.

**Table 2:** Non-invasive biomarkers (miRNA, Proteins, lnc RNA and circ-RNA) used for CRC detection till date.

Circulating nucleic acids and proteins in CRC				
Sample	miRNA	Protein	lncRNA	Circ-RNA
**Plasma**	miR-125a-3p, miR-193a-5p, miR-320c, miR-23b, miR-27a, miR-760, miR-130a, miR-29a, miR-210-3p, miR-92a, miRNA-18a, miR-100, miRNA-19a, miR-30e, miRNA-335, miR-16, miRNA-29a, miR-144-5p, miRNA-15b, let-7i, miRNA-19b, miR-486-5p, miR-20a, miR-181a-5p, miR-155, miR-30d-5p, miR-21, miR-24, miR-92, miR-29b, miR-106a, miR-194, miR-200c, miR-320a, miR-372, miR-375, miR-96, miR-423-5p, miR-92a, miR-601, miR-221, miR-760, miR-182, miR-320d, miR-506, miR-7, miR-4316, miR-93, miR-223, miR-31, miR-1290, miR-181b, miR-431, miR-203, miR139-p3, miR-139-3p, miR-409-3p, miR-18a, miR-22, miR-25, miR-29, miR-19a, miR-19b, miR-15b, miR-29a, miR-335, let-7g, miR-15b-5p, miR-18a-5p, miR-29a-3p, miR-335-5p, miR-19a3p, miR-19b3p	CPNE3 CEA Melanotransferrin	LNCV6_116109 LNCV6_98390 LNCV6_38772 LNCV_108266 LNCV6_84003 LNCV6_98602 91H PVT-1 MEG3 ATB CCAT1	circ-133 circPACRGL circ-ABCC1 circ_0000338 ciRS-122 hsa_circ_0004585 circ-FBXW7
**Serum**	miR-17-92a, miR-99b-5p, miR-19a, miR-150-5p, miR-1229, miR-548c-5p, miR-25-3p, miR-638, miR-17-5p, miR-33a-5p, miR-92a-3p, miR-210-3p, miR-135a-5p, miR-208b, miRNA-21, miR-139-3p, miRNA-31, miR-145, miRNA-92a, mir-92a, let-7g, miR-143, miRNA-181b, miR-21-5p, miRNA-203, miR-21, miR-96, miR-221, miR-139a-5p, miR-196b, miR-338-5p, miR-210, miR-1290, miR-103, miR-720, miR-106a, miR-17-3p, miR-92, miR-125, miR-223, miR-20a, miR-150, let-7a, miR-4516	FOXD2-AS1, QSOX1, NRIR, PKM2, LOC_009459, NNT-AS1, H19, CCAL, UCA1, HOTTIP, PrP(C), CA11-19, MIC-1 (GDF15), IL-6, IL-8, Growth-related gene, product β1, Cyr61, B6-integrin, TIMP-1, RBP4, THBS2, TFF3, COL3A1, COL10A1, AZGP1, Angiopoietin-2 7, Kininogen	CCAT1 UCA1 HOTAIR LOC285194 Nbla12061 RP11-462C24.1 BLACAT1	circ_0004771 circFMN2
**Stool**	miR-21, miR-29a, miR-135, miR-224, miR-92a, miR-7, miR-938, miR-222, miR-146a, miR-143, miR-138, miR-127-5p, miR-29b, miR-9, iR214, miR-199a-3p, miR-196a, miR-183, miR-17, miR-20a, miR-96, miR-106a, miR-134, miR-135b, miR-221, miR-18a, miR-223, miR-451, miR-144, miR-17-3p, miR-135b-5p, miR-421, miR-27a-3p	Haemoglobin (FIT) M2-PK MMP 9		

**Table 3:** Non-inavsive Protein and miRNA Panel used for CRC detection.

Sample	Protein Panel	miRNA panel
**Serum**	RBP4 and CEA TFF3 and CEA sDC-SIGN and sDCSIGNR IGFBP-3 and CEA AZGP1, CEA and CA19-9 IGFBP2, DKK3 and PKM2 CEA, hs-CRP, CYFra21-1 and Ferritin	miR-23a-3p, miR-27a-3p, miR-142-5p, miR-376c-3p Let-7a, miR-1229, miR-1246, miR-150, miR-21, miR-223, mir23a miR-19a-3p, miR-21-5p, miR-425-5p miR-301a, miR-23a miR-20a, miR-486 miR-223, miR-92a
**Plasma**	BAG4, IL6ST, VWF, EGFR and CD44	miR-103a-3p, miR-127-3p, miR-151a-5p, miR-17-5p, miR-181a-5p, miR-18a-5p, miR-18b-5p miR21, miR25, miR18a, miR22 miR-1290, miR-320d
**Stool**	Complement C3, Lactotransferrin, Haemoglobin subunit α1 and Haptoglobin	(miR-17, miR-18a, miR-19a, miR-19b, miR-20a, and miR-92a miR-144-5p, miR- 451a miR-20b- 5p

## Mitochondrial DNA: Unexplored arena

In the recent era, the standard for the molecular profile of colorectal cancer (CRC) is tissue biopsy. However, they are inadequate concerning about sampling rate, illustration of tumor heterogeneity, and sampling can expose patients to antagonistic side effects. To study cell-free DNA (cfDNA) from the various body fluids, this being a component of a liquid biopsy, is relatively invasive, but highly significant to discover all tumor-specific mutations. Furthermore, mitochondria have their circular genome and therefore contribute to the total cfDNA content in the blood. MtDNA plays an essential role in mitochondrial biogenesis and regulates mitochondrial function and the regulation of apoptosis (180–182). A single cell comprises up to several thousand copies of mitochondrial DNA (mtDNA) contrasting to two copies of nuclear DNA (nDNA). Therefore, investigating hypothetically cell-free mitochondrial DNA (cf-mtDNA) could give an advanced level of understanding, rather than the examination of cell-free nuclear DNA (cf-nDNA). Furthermore, it was reported that mtDNA has a high mutation frequency and in CRC and other cancers fundamental molecular modifications (183, 184). Based on reported research literature, assessment of cf-mtDNA as a significant biomarker is stimulating for liquid biopsies and as a neoantigen due to high copy number might enable discovery of even minor quantities of ctDNA and their molecular modifications. Besides, earlier research has exposed that cf-mtDNA content and fragmentation design distinguish between cancer patients and healthy individuals, therefore also potentially serving as an indicative marker of disease (185–188). Though cf-mtDNA has been not completely categorized yet and an efficient method for comprehensive examination is still missing.

Rigorous research has been done to understand the hereditary risk issues of CRC. Thus far, over 40 nuclear genome alternatives significantly related to CRC risk have been recognized, counting SNP rs10911251, rs1321311, rs1035209, and so on (189, 190). But such loci account for only about 8%–16% of CRC cases, signifying that additional genetic risk factors of CRC still possibly need to be discovered.

Remarkably, numerous somatic mtDNA mutations and copy number alterations have also been commonly recognized in a wide variety of malignancies, including CRC (191). In CRC it was observed that mtDNA copy number is increased during the cancer process. Previous studies by Guo et al. have described that the reduction of mtDNA made by the mitochondrial transcription factor A (TFAM) mutation plays a potential role in cancer progression and resistance to cisplatin in MSI CRC (192). In addition to this, a report from China investigated 104 colorectal cancer patients and found that the percentage of mtDNA deletion of 4977 bp of mtDNA in CRC tissues was significantly reduced (193). Furthermore, recent research revealed that mitochondrial cfDNA had a surprisingly higher plasma copy number in healthy subjects than in CRC patients (188). Though, today the possible contribution of germline mtDNA differences in CRC expansion is a smaller amount of knowledge available including liquid biopsy. Together, we are confident that liquid biopsy is likely to be a substitute standard approach for monitoring the advanced development of genomic changes during cancer progression. Liquid biopsy has revealed remarkable effectiveness in a variety of applications and will contribute to personalized oncology.

## Organoids

Tumor organoids were reviewed by Tatullo et al. with 77 references (194). To date, scientific cancer research has been conducted in *in vitro* experiments, performed on tissue culture plates and two-dimensional (2D) samples. In this framework, the development of colonies and spheroids has been determined as morphological indicators of cancer and stemness of cancer cells (195). In the current research scenario, 3D cultures systems have significantly enhanced *in-vitro* tumor models based on new biological mediums that mimic the extracellular environments. Organoids have been described more extensively in many reports in the scientific research literature. The overview of patient-derived organoids (PDO) has allowed for more representative cancer modeling, highlighting their excessive significance in biomedical applications, translational medicine, and personalized therapy approaches (196, 197). Furthermore, patient-derived organoids have certain advantages such as stable morphology, gene expression, and cell signaling, heterogeneity with cancer cells in the tumor, significant drug screening, low cost, and being easily generated “in a dish” (198). The application of the organoid culture method to liquid biopsy is a promising approach that combines the advantages of organoid cultures with the boundless potential of the liquid biopsy component for precision oncology.

Sato and their team first developed an organoid model from mice in the CRC research field and later they also developed an organoid culture protocol that is acceptable and also suitable for colon epithelial cell culture (199). In CRC, PDO developed from metastases taken by serial biopsies at various time points, and various counties of severely pretreated CRC patients were taken as preclinical models in clinical trials studies (200, 201). Those organoids were further treated with anti-cancer drugs, and the outcomes were associated with patients’ responses in clinical trial studies. The outcome suggested the ability of PDO to mimic TME *in vivo*, notable molecular and functional levels, and the most important aspect being to predict patient treatment response (202). Clinically active KRAS signaling suppressors and various drug groupings were observed against non-cancerous colon and CRC organoids (203). In recent research Zhao et al. used the organoid culture approach to identify the metabolic phenotype in cancer stem cells and differentiated cancer cells in CRC (204). To date, only one study has been done on organoids derived from CTCs and it revealed that CTC-derived organoids were more sensitive than Xenograft-derived organoids, to drugs affecting the Survivin pathway, which significantly decreased the levels of Survivin and X-linked inhibitor of apoptosis protein (XIAP), that induce CTC derived organoid death. Based on this first study, future use of the organoid approach to CTCs may open new viewpoints by providing extraordinary visions of the cancer growth and metastatic process, by allowing the discovery of novel CTC markers, beneficial treatment targets, and chemoresistance mechanisms (205).

Notwithstanding organoid significant advantages, patient-derived organoid (PDO) also possesses certain limitations such as abnormalities, noise during drug screening, development and standardization of organoid culture, and lack of major TME components (206–208). Based on the published literature, PDO is a fascinating *in vitro* model for the development of preclinical drugs in CRC, because of its ability to mimic human physiopathology. Taken together, the potential of the organoid approach for basic and clinical studies of CRC is greater than the treatment of patients with CRC in the new time of personalized medicine. Furthermore, it will open a new door for the liquid biopsy approach using CTCs and/or CTSCs to generate organoid models.

## Liquid biopsies and immunotherapy

In the research of CRC treatment, diagnostic and chemotherapy have developed curiously in the last two eras. Still, it is problematic to find minimal residual disease (MRD) essential for primary detection of recurrence of tumors and give suitable drugs timely prior cancer becomes multi-drug-resistant and more aggressive. However, the most thrilling example of change in cancer therapy in the current era has been immunotherapy. Subsequently, with its early approval for the treatment of melanoma, it has become the standard of care for various other tumors. Immunotherapy has also established promising abilities and good tolerance in gastrointestinal (GI)-related cancers (209). All the research conferred so far in CRC are focused either on the association between ctDNA existence and tumor burden or the recognition of molecular modifications that predict response or resistance to targeted agents. The burden of tumor mutations is currently being argued in CRC and various solid tumors were given its association with response to immunotherapy and the current approval of the Food and Drug Administration (FDA) as an agnostic biomarker to access cancer immunotherapy with pembrolizumab or dostarlimab (210, 211). On the other hand, MSI is currently the most applicable potential biomarker for immunotherapy sensitivity in CRC, characteristically measured in solid tissue samples (212). Additional growing manipulation of liquid biopsy in CRC is the examination of methylation biomarkers, which is rapidly developing as an influential approach to early diagnosis and prognosis (213).

### MSI colorectal cancer

Microsatellite instability (MSI), also known as a hypermutable phenotype, occurs because of a defective mismatch repair system (dMMR) in approximately 15% of colorectal cancer patients (CRC) (214–216). MSI CRC is most often associated with the proximal colon, increased immunogenicity, and a good prognosis, in contrast to CRC of chromosomal instability (CIN) (also known as stable/low-level microsatellite stable/MSI-low-level [MSS/MSI-L]) CRC which is more commonly found in the distal colon with increased immune tolerance and a poor prognosis (215, 217). Many studies have shown the advantages in detecting MSI status, including prognosis and specific treatment benefits associated with this molecular subtype, with increased survival rates of up to 15% in CRC patients (218, 219). A few studies thus far have illustrated MSI to be a rare occurrence in rectal cancer, and linked to a poorer prognosis with a higher risk of dying (220–222). Better results are observed in locally advanced (stage II/III) MSI CRC compared to CIN CRC, with the recently added benefit of oFDA-approved immunotherapy (i.e. pembrolizumab, nivolumab, and combination nivolumab/ipilimumab) in the treatment of unresectable or metastatic resistant MSI CRC in conventional regimens (223–226). To date, the conventional treatment regimen for rectal cancer continues to be resection surgery, chemoradiation (preoperative), and chemotherapy, with the intolerant response that do not have alternative approved treatment strategies available (227, 228). MSI CRC is known to have a poor response to 5-fluorouracil (5-FU), which is a fluoropyrimidine drug used in the conventional adjuvant treatment regimen of CRC (229). Adverse effects include nausea, diarrhoea, mucositis, neuropathy, neutropenia and more serious complications leading to death have been reported in 1% of patients. Therefore, it is imperative to implement a reliable diagnostic methodology for accurate diagnosis of MSI. Mononucleotide markers have been well described as the most reliable markers for MSI panels, without the need for di-nucleotide markers and matched normal tissue testing (230, 231). Ethnic polymorphisms have also been described in certain markers (eg. African polymorphisms in BAT25 and BAT26) and should therefore be considered when deciding on the implementation of diagnostic markers panel in certain geographical settings (231–233). If instability is required in 30% of markers used in the panel for a diagnosis of MSI, it is important to establish that the markers included are nonpolymorphic in the general population. Additional testing to confirm MSI status is to assess the expression profiles of mismatch repair (MMR) proteins through immunohistochemistry (IHC) (234–236). This is a more cost-effective approach and in addition provides information on the deficient dMMR protein, gaining insight into the possible mechanism of the disease, whether likely sporadic (associated with MLH1 protein loss through MLH1 promoter methylation, and BRAFV600E pathogenic variants) or due to hereditary Lynch syndrome pathogenic variants (MSH2, MSH6, MLH1, or PMS2) (237–239).

MSI CRC is known to have a better response to immunotherapy, and this is due to the active innate inflammatory tumor microenvironment, as a response to the hypermutated phenotype of these tumors (240). The TCGA study revealed that hypermethylated and hypermutated cancers were more commonly associated with the proximal colon and distinct at the genomic level compared to distal colon and rectum cancer (217). This could potentially be due to the difference in the originator cells of the right colon (developed from the midgut) compared to the distal colon (originated from the hindgut) (241, 242). To date, few clinical trials have also begun exploring combination radioimmune therapy, with promising toxicity reports indicating hope for patients with rectal cancer (243). Another remarkable study of a PD-1 blockade (dostarlimab) in the treatment of MSI rectal cancer indicated high sensitivity and 100% complete response rates with no severe adverse events (244). This illustrates the need for more clinical trials in immunotherapy and neoadjuvant therapy with a focus on rectal cancer to be conducted, to provide more effective predictive therapy for the better management and increased survival of these patients. Besides this, the neoantigens currently appear in MSI-H CRC, which is related to a higher tumor mutation burden, so it has potential as neoantigens in the immunotherapeutic strategy for the treatment of various types of CRC. But a liquid biopsy-based examination to assess MSI can successfully assess an extensive subclass of CRC patients, including those with inadequate tissue samples or when protection concerns about invasive surgery arise.

### MSS colorectal cancer

Tumors in the distal colon display lower mutational burdens and are less immunologically active, with little to no CD8^+^ T lymphocyte localization or infiltration. This type is generally referred to as a “cold-tumor” (245). Cold-tumors represent the majority of CRC and mostly do not benefit from immune checkpoint inhibitor (ICI) therapy. Improvement in immune therapeutic strategies includes transitioning “cold-” into immune infiltrated “hot-tumors”, and once infiltrated, ensuring an effective inhibitory response on tumor cell activity is attained (245). This is achieved by controlling tumor immunogenicity and the TME by directing the immune system in targeting tumor cells specifically (246). ICIs are designed to inhibit certain receptors such as Programmed-death-1 (PD-1) on T-cells that are controlled by cancer cells to evade immune attack. Monoclonal antibody (mAb) treatment, chimeric antigen receptor (CAR)-T cell therapy, and ICIs are key immunotherapies currently being used against many cancers (247). mAb therapy against the receptor Programmed death ligand-1 (PD-L1) on cancer cells, to block its communication with PD-1 and increase T cell immune response has shown effective in many solid tumors. Adoptive cell transfer (ACT), such as chimeric antigen receptor (CAR) T-cell therapy involving the patient’s own T-cells has also gained increased recognition (248). These cells are genetically engineered to include the new CAR, and then re-administered to the patient (247) The CAR increases-affinity and binding of T-cells to target antigens, without the need of the major histocompatibility complex (MHC) receptor. CAR-T therapy has had fewer success rates in solid tumors, mainly due to a suppressive TME (increased cytokine and dense stromal network) (248). Enzymes targeting and degrading stromal matrices (eg. heparanase) have been employed to overcome this hurdle and increase infiltration of CAR T-cells in solid tumors (249, 250). Cancer vaccines have also been introduced as novel immunotherapeutic approaches to target antigens uniquely expressed on tumor cells, thus inducing an anti-tumor immune response in patients (251). In addition, oncolytic viruses destroying cancer cells but non-virulent to normal cells is another immunotherapy strategy (247). Certain virus, have natural tropism to infect certain cells, for example, hepatitis B virus for hepatocytes and parvovirus B19 for human erythroid progenitor cells, and this mechanism has been used to direct virus-mediated cytotoxicity in tumor cells (252). To address effective immunotherapeutic strategies in MSS CRC in future, combination therapy involving two or more approaches would need to be implemented, involving chemotherapy, radiotherapy, mAb, ICI targeted therapy, stromal matrix degradation, oncolytic viral therapy, CAR-T therapy and cancer vaccines (247).

### Neoantigen: An emerging concept

Neoantigens have potential high specificity and targeted but are mainly patient-specific and, consequently, are difficult to classify for utility and are mostly remarkable procedures in a cancer patient population. Currently, immunotherapy, inclusive of immune checkpoint inhibitors (ICIs), tumor-specific vaccines, and tumor-infiltrating lymphocytes (TILs) based on neoantigens, has a progressively significant role in cancer treatment (253). The conventional significant cDNA library screening method is labor-intensive, low-throughput, and unable of classifying some altered antigens consequent from GC-rich transcripts and low-expression transcripts (254). However, current scientific developments in next-generation sequencing and a notable improvement in bioinformatics analysis have provided a robust groundwork on which to build these significant efforts. A peptide-based identification method connecting whole-exome sequencing (WES) and MHC-peptide binding prediction algorithms has been effective in identifying neoantigens recognized by TILs in patients with melanoma (255). Neuropeptides are expressed in tumor cells, while healthy cells will not present such antigens. Earlier research on CRC genomics mostly focused on the mechanism of tumor development and progression, with a lower inclusion of neoantigens and neoantigen-based immunotherapy (256). In research, it was observed that certain CRC patients with high microsatellite instability (MSI-H) might benefit from ICI treatment due to the presence of high neoantigens (256). However, not all patients with MSI-H CRC show medical efficacy in ICI treatment. Neoantigen-based immunotherapy is synchronizing with ICI since it does not need a detailed analysis of the patient’s MSI status or tumor mutation burden (TMB) (257). The tumor-specific landscape of neoantigens makes them significant perfect targets for antitumor immunotherapy and has been investigated for the treatment of CRC in a variety of basic and clinical immunotherapy studies. The average TMB of CRC was classified seventh among 30 of the most common categories of tumors. A previous study by Aleksandrov L. B observed that approximately 16% of CRCs have a TMB of >12 mutations per 106 base pairs, which are identified as extremely mutated tumors (258). Patients with higher TMB might have more potent neoantigens that can be used for the clinical approach in CRC. For MSI-H CRC, frameshift mutations generally instigated by INDELs can lead to the creation of novel frameshift peptides (FSP), which are the key cause of neoantigens in CRC. Frameshift mutations can be frequently initiated in DNA segments or genes with a significant biological role in maximum MSI-H CRC. These genes play a vital role in epigenetic regulation, DNA repair, signal transduction, cell apoptosis, and miRNA processing. Besides frameshift mutation currently, it has been described that single-nucleotide variants (SNVs) in genes like KRAS, PIK3CA, PCBP1, and CHEK2, are related to the creation of the 10 most frequent neoantigens. In **Table 4**, we have listed the mutated antigens that were studied in CRC tissue (259).

**Table 4:** List of mutated antigens found in CRC.

Frameshift Mutation Genes	SNVs Genes
OGT	KRAS
TGFβRII	PIK3CA
BAX	PARVA
MSH3	G3BP1
FTO	ACTR10
Caspase 5	RAE1
	PDP1
	QRICH1

One main hurdle for personalized neoantigen-based immunotherapy is the availability of tumor biopsies. To date, neoantigens are usually recognized from genomic profiling of various tumor biopsies (260). Although this predictable approach is time-consuming, invasive, with a low positivity rate, and in the most challenging case where repeated sampling is mandatory or there is an inadequate sample, it is more common with frequent and metastatic cancers. Specifically, at the top immune checkpoint, significant inhibitors can be more effective in the presence of natural neoantigens (261, 262). Based on the current scenario, liquid biopsies can be a good replacement for determining potential neoantigens as budding targets for immunotherapy in numerous cancers. Although there is a certain restriction in the detection of genomic mutations with very low allele occurrence in the plasma sample, the dependability of genetic information has been described concerning the use of liquid biopsy (263). Thus, based on current research on liquid biopsies, valuable visions could be served for making treatment choices using neoantigen.

### Immune cells

**Figure 6:**
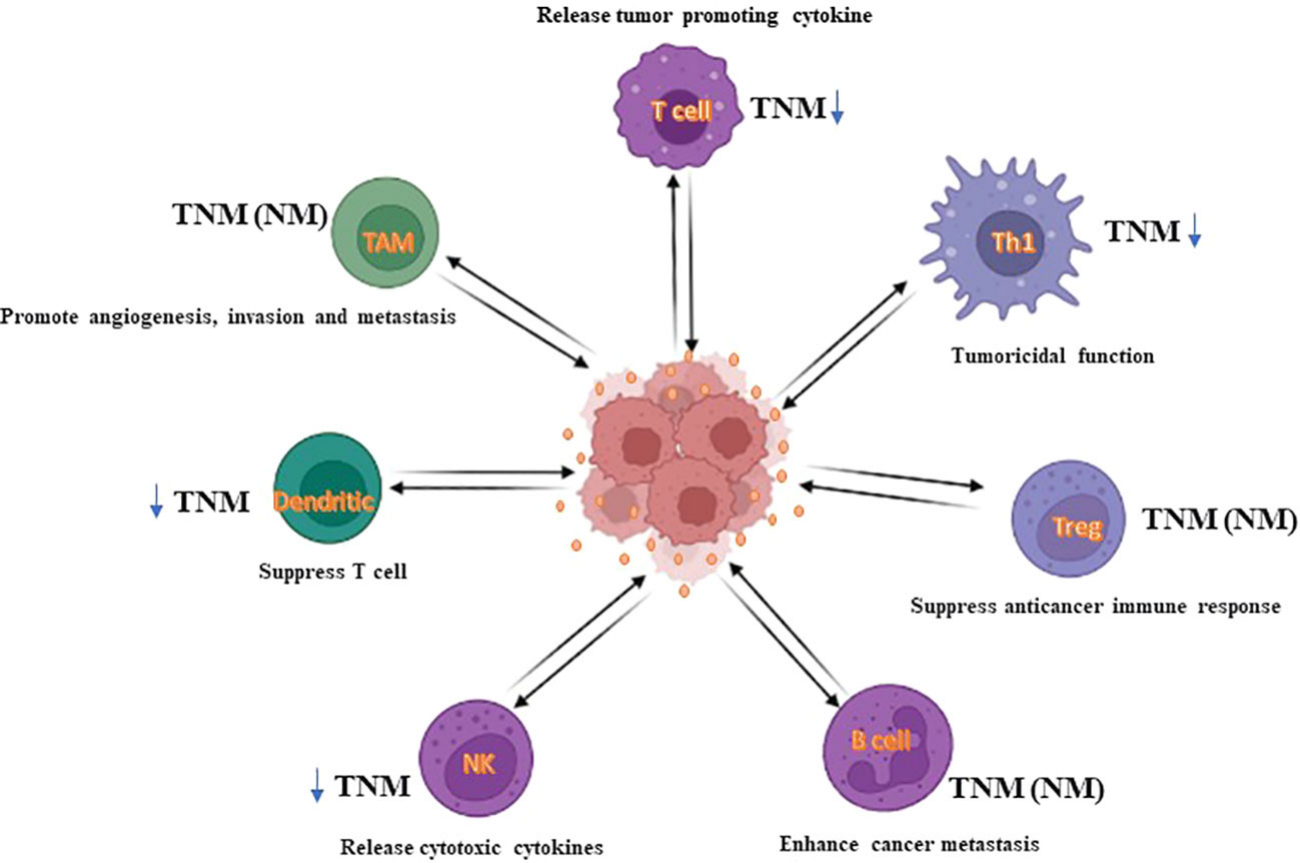
The impact of immune infiltrates on CRC. In CRC, immune infiltrates can impact CRC cell death, either directly or *via* tumoricidal T cells (TCT), and consequently affect tumor progression. For example, cytotoxic T cells, macrophages, and NK cells can exert a cytolytic effect on CRC cells. For other populations of cells, such as Treg, B cells, dendritic cells, or M2-like macrophages, generally impact CRC cell death by mediating the tumoricidal activity of TCT cells. Herein, Treg, regulatory B cells, immature dendritic cells, and macrophages enable TCT cells to be exhausted, thus causing substantial progression in CRC tumors. Accordingly, immunoscore system using immune cells could deliver insights into a novel liquid biopsy approach as a diagnostic tool.

The TME generates a potential protective shell in which cancer cells easily and rapidly gather gene alterations and immune escape. Generally, this process occurs in the early stage of cancer, the immune response created by immune cells in the TME has antitumoral properties (264). Collectively, evidence has revealed that TME contains NK cells, CD8^+^ cytotoxic T cells, M1 macrophages, T helper-1 cells, and antigen-presenting cells (APCs) which act as tumor foes and suppress tumor development. Neutrophils, tumor-associated macrophages (TAMs), CD4^+^ T helper-2 cells, and regulatory T cells (Tregs) are crucial components for reducing the immune suppression environment, inhibiting cancer cell survival and progression, in addition to helping to avoid immune devastation (265) (**Figure 6**). In metastatic CRC, it has been confirmed that tumor behavior with the lowest tumoricidal immune infiltrates shows a higher risk of tumor replacement (266). CD8+ T and CD4+ T cells are the utmost powerful cytolytic cell subcategory. Cytotoxic processes are supported by some constituents shaped by CD8+ T cells, such as granzymes, perforin, Fas ligand (FasL), and TNF-α (267). Recent research established that patients with promising CRC regularly have tumor immune cell infiltrates with higher cytolytic events (268). But, the percentage of cytotoxic T cells number decreases as TNM-stage increases in CRC (269).

In humans, Treg cells are the main source of IL10. IL10 has numerous effects on immune cells, including decreasing the cytotoxic activity of CD8+ T cells, down-regulating MHC-II-restricted antigens, preventing the synthesis of IFN-γ or TNF-α, and hindering the effector roles of dendritic cells and other CD4+ T cell subsets (Th1, Th2, or Th17 cells) (270, 271). Based on the results of numerous reviews, there is still support that tumor infiltration of Treg cells possibly extends the survival of CRC patients (272). In concept, Treg cells are susceptible to apoptosis in CRC tumors that could negatively regulate the expression of IFN-γ, TNF-α, and IL-2 by tumoricidal T cells (273). Collectively this suggested that the concentration of Treg cells along with their connected cytokine profiles in cancer should be determined together in a liquid biopsy-based approach to increase the use of Treg cells in predicting CRC prognosis.

B cells consist of diverse subcategories and govern antibody production, antigen appearance, and immunosuppression (274). A currently published study on colon cancer has reported that a high concentration of tumor B cells may provide for promising clinical outcomes only in patients with right-sided colon cancer (275). Furthermore, the higher expression of CXCL9 and CXCL10 in CRC tumors can also attract regulatory B cells (Breg), although such chemoattractants are also effective in employing tumoricidal T cell functions (276). Assessing the concentrations of tumoricidal T cells, Treg cells, and B cells together could significantly improve the prediction of the prognosis of CRC. In addition to this component, natural killer cells (NK) also play a cytolytic role in TME. In CRC, it was found that alteration of MHC-I functions, resulting in NK cells, will reduce its development and decrease the production of IFN-γ, GZMB, and perforin production (277). Surprisingly, in CRC metastasis, it was observed that the number of tumor-formed NKT cells was markedly decreased compared to normal tissue (278). However, it is at minimum knowledge that NK cell infiltration into CRC at progressive disease phases is challenging. In TME, one of the most significant components of dendritic cells (DCs) is specialized antigen-presenting cells in the human body. Previous data suggested that in CRC tumor infiltration of DCs is negatively related to tumor phases because this growth of DC cells with various phenotypes will result in a poor prognosis of CRC (279). Fundamentally, it is indicated that mature or immature DC could have various effects on CRC development. Lastly, the major component of Tumor-associated macrophages (TAMs) are dangerous immune infiltrates in cancer phenotype. In CRC, numerous studies have shown that a high number of CD68+ macrophages in tumor IM expect a promising prognosis (280, 281). Furthermore, Itatani et al. observed that by improving the production of metalloproteinase-9, CCR1+ macrophages support the invasion of CRC (282). Similarly, to CCL2 and CCL15, CCL5 helps as another significant chemokine that controls the development of CRC (283). Besides, in CCL5-deficient mice, xenografted CRC tumors show a high amount of tumoral CD8+ T cells, signifying that CCL5 at minimum influences T cell infiltration (284).

The Immunoscore system delivers insights into a novel approach for consistently predicting CRC diagnosis, particularly since this tool has the potential to screen immunotherapy components. On the other hand, Immunoscore combined with diagnostic tools such as liquid biopsy, and a neoantigen-based approach provides for better CRC treatment, especially for immunotherapy.

## Future perspective and conclusion

The prognosis of individuals with CRC has substantially improved in the current era due to the significant improvement and expansion in diagnostic and therapeutic approaches. However, early prediction, diagnosis, and treatment monitoring of CRC have various lacunae; due to this, many patients die each year. In recent years, the field of liquid biopsy has grown rapidly because it is noninvasive, overcomes tumor heterogeneity, and can allow real-time intensive care of tumor development, recurrence, or therapeutic response (285). This is the reason that recently there are numerous ongoing clinical trials from the US National Laboratory of Medicine (NIH) on liquid biopsy-based approaches to detect CRC. Presently, numerous efforts have been made utilizing CTCs, CAFs, exosomes, immune cells, neoantigens, mtDNA, and ctDNA isolation and characterization-based approaches to detect and treat CRC; and which have shown to be highly sensitive and effective. In addition, genes and proteins expressed by these components can also be used for early CRC detection and therapy. However, a CTC end point value for the clinical evaluation of CRC patients’ progression and prognosis is still not adequately developed owing to sampling issues, storage conditions and timing of biopsy; and most importantly enrichment procedures (286, 287). Therefore, it is important to develop a CTC capturing platform that is more precise and effective. Additionally, recent studies on CTCs/cCAFs clusters open a new path for developing an additional personalized and detailed treatment plan for each cancer patient. But there are still several lacunae on the biology of CTCs clusters, and specifically on the heterotypic CTCs-CAFs clusters, that need to be investigated to recognize the mechanism of cellular aggregates and their role in metastasis. Furthermore, it is also important to see which of the CAF-derived signals improve CTC survival and cancer cell growth, besides to govern the efficient alterations between homotypic CTCs clusters and heterotypic CTCs-CAFs clusters. Another significant component of liquid biopsy is the exosome, that has a potential role in tumor initiation, development and metastasis, including EMT, tumor angiogenesis, extracellular matrix remodeling, organ-specific metastasis, and immune evasion. The advantage of exosomes is that they are easier to isolate than CTCs and cfDNA in tumors; a current era is improved and more research will be focused on exosomes in the diagnosis of cancers at an early stage in the future. But there is still uncertainty in clinical approaches due to low effectiveness and informal phagocytosis by the immune system. So, based on evidence, indepth research should be undertaken to solve this hindrance and develop precise clinical applications of exosomes. Furthermore, analysis of ctDNA is a most promising component of liquid biopsy that can play a critical role in numerous characteristics in the clinical management of patients with CRC (288). Furthermore, TMB in ctDNA and immune check point proteins in CTCs show significant roles in tumor immunotherapy. However, due to inadequate and partial knowledge of molecular mechanisms, ctDNA as liquid biopsy has not yet been applied in immune-oncology in the clinic; however, promising available data and advanced noteworthy technologies and methods recommend that this approach certainly has a plausible role in CRC patient therapy. Based on our review, we found that a higher copy number of mtDNA significantly promotes cell proliferation, apoptosis resistance, and CRC metastasis, thus also providing a novel indication for this process as a drug target and prediction of neoantigens in CRC treatment (188). Existing genomic research has revealed that there are many hotspot mutations in significant driver genes; and the neoantigen epitopes made by these mutations are vital “public” immunotherapy targets as a liquid biopsy approach. More recently, liquid biopsy-based neoantigens are a new immunotherapeutic approach for the treatment of various types of CRC. Though, there are still numerous challenges such as tissue biopsy and identification, which still require further research as explored form of liquid biopsy (**Table 5**). The worldwide replacement of tumor biopsies with liquid biopsies appears idealistic; however, with a range of approaches using CTCs, CAFs, ctDNA, exosomes, mtDNA and neoantigen, it seems highly likely that useful tools will be developed for CRC with applications in early detection, postoperative monitoring, treatment response and therapeutic resistance. In summary, liquid biopsy is an important part of precision medicine and is held to be a clinical reality soon.

**Table 5:** Advantages and disadvantages of liquid biopsy components.

**Component**	**Advantage**	**Disadvantage**
**ctDNA**	Well established methods for detection of tumor-specific genetic abnormalities with greater sensitivity Analyze cancer origin and prediction of drug effectiveness Detection of acquired resistance and/or minimal residual disease Cancer progression and metastasis monitoring	Unsuitable for functional test due to impaired detectability (low ctDNA abundance) Background noise from typical cell-free DNA Difficulties in standardizing procedures
**mtDNA**	Compared to nuclear DNA, single cell contains several thousand copies of mtDNA Higher sensitivity Enable detection of even small amounts of molecular alterations due high mutation rate Potential prognostic marker due to differential fragmentation pattern between cancer patients and healthy individuals	Not fully characterized yet Lack of optimized protocol for cf-mtDNA Large scale prospective studies are needed
**CTCs**	Feasible for molecular and morphological identification Possible prognostic and/or predictive markers for monitoring cancer progression and metastasis Potential therapeutic targets Useful for *in-vitro* culturing to test drug sensitivity DNA, RNA and protein profiling	Low specificity- especially in early stage settings Difficulties with detection method standardization due to EMT and heterogenous biomarkers for identification Short half-life
**cCAFs**	Well established role in cancer progression and metastasis Advantage survival in circulation by forming clusters with CTCs and/or CSCs Potential therapeutic target Detection and monitoring of minimal residual disease Potential biomarker for early detection and prognosis Prospective model for better understanding of TME	Larger confirmatory studies are needed Lack of robust and standardized methods for detection

## Author contributions

SM and CP conceptualized and designed the manuscript. SM, KB and MM contributed in literature review and drafted manuscript. MJM created all figures. SM, KB, RS and CP contributed to critical review and finalized the manuscript. All authors contributed to manuscript and approved the submission.
